# Draft Genome Sequence of a Hypersensitive Reaction-Inducing *Pantoea agglomerans* Strain Isolated from Olive Knots Caused by *Pseudomonas savastanoi* pv. savastanoi

**DOI:** 10.1128/genomeA.00774-14

**Published:** 2014-08-07

**Authors:** Chiaraluce Moretti, Chiara Cortese, Daniel Passos da Silva, Vittorio Venturi, Emanuela Torelli, Giuseppe Firrao, Roberto Buonaurio

**Affiliations:** a Dipartimento di Scienze Agrarie, Alimentari e Ambientali, Università degli Studi di Perugia, Perugia, Italy; b International Centre for Genetic Engineering and Biotechnology, Trieste, Italy; c Dipartimento di Scienze Agrarie e Ambientali, Università degli Studi di Udine, Udine, Italy

## Abstract

*Pantoea agglomerans* strains inducing a hypersensitive reaction in tobacco leaves are frequently isolated inside olive knots caused by *Pseudomonas savastanoi* pv. savastanoi. Here, we report the draft genome sequence of the Italian *P. agglomerans* strain, which is able to increase olive knot disease severity when coinoculated with *P. savastanoi* pv. savastanoi.

## GENOME ANNOUNCEMENT

Many bacterial species live inside olive knots caused by *Pseudomonas savastanoi* pv. savastanoi and belong mainly to the genera *Pantoea*, *Pectobacterium*, *Erwinia*, and *Curtobacterium* (1). From olive knots, we have frequently isolated *P. agglomerans* strains inducing a hypersensitive reaction (HR) in tobacco plants, and we demonstrated that one of them (DAPP-PG 734) (i) forms a stable interspecies community with *P. savastanoi* pv. savastanoi, (ii) communicates through a quorum-sensing system mediated by *N*-acyl-homoserine lactones, and (iii) increases disease severity when coinoculated with the pathogen in olive plants (2, 3). To better understand the molecular basis of the interaction between *P. savastanoi* pv. savastanoi and the *P. agglomerans* DAPP-PG 734 strain, we sequenced the genome of this strain isolated in Italy from an olive knot.

Genomic DNA was prepared for sequencing by the Nextera DNA sample preparation kit (Illumina), according to the manufacturer’s instructions. Sequencing was performed on an Illumina MiSeq platform using indexed paired-end 250-nucleotide v2 chemistry. The Edena assembler (4) was used to assemble 5.3 Mbp, representing approximately 37-fold coverage of the genome and comprising 195 contigs, with a maximum length of 172 kbp and an *N*_50_ of 53 kbp, assuming a genome size of 5.3 Mb. The G+C content was 54.6%, which is similar to that of other sequenced *P. agglomerans* genomes.

Automated annotation of the *P. agglomerans* draft genome sequence using RAST (5) assigned a total of 3,697 candidate protein coding-genes, with 1,357 (26.85%) annotated as hypothetical proteins. The assembly predicted a total of 71 tRNA and 36 rRNA sequences.

The HR induced by the strain seems to be explained by the presence of a complete *hrp*/*hrc* gene cluster showing remarkable synteny and high sequence similarity with *Erwinia amylovora* and *Erwinia pyrifoliae* homologs. Mutants of the *hrpN*, *hrpY*, and *hrpJ* genes were obtained, and the determination of their phenotypes will be described in a future publication.

### Nucleotide sequence accession numbers.

This whole-genome shotgun project has been deposited at DDBJ/EMBL/GenBank under the accession no. JNVA00000000. The version described in this paper is version JNVA01000000.
